# Treatment of sporadic Burkitt lymphoma in adults, a retrospective comparison of four treatment regimens

**DOI:** 10.1007/s00277-017-3167-7

**Published:** 2017-12-06

**Authors:** L. E. M. Oosten, M. E. D. Chamuleau, F. W. Thielen, L. C. de Wreede, C. Siemes, J. K. Doorduijn, O. S. Smeekes, M. J. Kersten, L. Hardi, J. W. Baars, A. M. P. Demandt, W. B. C. Stevens, M. Nijland, G. W. van Imhoff, R. Brouwer, C. A. Uyl-de Groot, P. M. Kluin, D. de Jong, H. Veelken

**Affiliations:** 1 Present Address: Department of Immunohematology and Blood Transfusion, Leiden University Medical Center, Albinusdreef 2, 2333 ZA Leiden, The Netherlands; 2 Department of Hematology, Leiden University Medical Center, Leiden, The Netherlands; 3 Department of Hematology, VU University Medical Center, Amsterdam, The Netherlands; 4 Erasmus School of Health Policy & Management / Institute for Medical Technology Assessment, Erasmus University, Rotterdam, The Netherlands; 5 Department of Medical Statistics and Bioinformatics, Leiden University Medical Center, Leiden, The Netherlands; 6 Department of Hematology, Erasmus MC Cancer Institute, Rotterdam, The Netherlands; 7 Department of Hematology, Academic Medical Center, Amsterdam, The Netherlands; 8 Department of Medical Oncology, Antoni van Leeuwenhoek Hospital, Amsterdam, The Netherlands; 9 Department of Hematology, Maastricht University Medical Center, Maastricht, The Netherlands; 10 Department of Hematology, Radboud University Medical Center Nijmegen, Nijmegen, The Netherlands; 11 Department of Hematology, University Medical Center Groningen, Groningen, The Netherlands; 12 Department of Hematology, Reinier de Graaf Hospital, Delft, The Netherlands; 13 Department of Pathology and Medical Biology, University Medical Center Groningen, University of Groningen, Groningen, The Netherlands; 14 Department of Pathology, VU University Medical Center, Amsterdam, The Netherlands; 15 Department of Internal Medicine, Deventer Hospital, Deventer, The Netherlands; 16 Department of Internal Medicine, Alrijne Hospital, Leiderdorp, The Netherlands

**Keywords:** Burkitt lymphoma, Drug therapy, Survival, Cost analysis

## Abstract

Burkitt lymphoma is an aggressive B cell malignancy accounting for 1–2% of all adult lymphomas. Treatment with dose-intensive, multi-agent chemotherapy is effective but associated with considerable toxicity. In this observational study, we compared real-world efficacy, toxicity, and costs of four frequently employed treatment strategies for Burkitt lymphoma: the Lymphome Malins B (LMB), the Berlin**-**Frankfurt**-**Münster (BFM), the HOVON, and the CODOX-M/IVAC regimens. We collected data from 147 adult patients treated in eight referral centers. Following central pathology assessment, 105 of these cases were accepted as Burkitt lymphoma, resulting in the following treatment groups: LMB 36 patients, BFM 19 patients, HOVON 29 patients, and CODOX-M/IVAC 21 patients (median age 39 years, range 14–74; mean duration of follow-up 47 months). There was no significant difference between age, sex ratio, disease stage, or percentage HIV-positive patients between the treatment groups. Five-year progression-free survival (69%, *p* = 0.966) and 5-year overall survival (69%, *p* = 0.981) were comparable for all treatment groups. Treatment-related toxicity was also comparable with only hepatotoxicity seen more frequently in the CODOX/M-IVAC group (*p* = 0.004). Costs were determined by the number of rituximab gifts and the number of inpatients days. Overall, CODOX-M/IVAC had the most beneficial profile with regards to costs, treatment duration, and percentage of patients completing planned treatment. We conclude that the four treatment protocols for Burkitt lymphoma yield nearly identical results with regards to efficacy and safety but differ in treatment duration and costs. These differences may help guide future choice of treatment.

**Electronic supplementary material:**

The online version of this article (10.1007/s00277-017-3167-7) contains supplementary material, which is available to authorized users.

## Introduction

Sporadic Burkitt lymphoma (BL) is a rare and highly aggressive B cell malignancy, accounting for 1–2% of all adult lymphomas in Western Europe and North America. In the adult population, BL most often affects young to middle-aged patients with a median age at diagnosis of 35 years [1]. Patients often present with bulky extranodal disease, bone marrow infiltration, and central nervous system involvement [2, 3].

Because of rapid tumor growth, prompt diagnosis and start of treatment are important to optimize outcome [4]. Current treatment strategies for adults have often been adapted from pediatric protocols [2, 3]. All of these protocols aim to deliver dose-intensive, multi-agent chemotherapy with minimization of treatment delays, and maintenance of serum drug concentrations. Examples are the French Lymphome Malins B (LMB) regimen developed by the Société Française d’Oncologie Pédiatrique [5, 6], the German Berlin-Frankfurt-Münster (BFM) regimen developed by the German Multicenter Study Group for Adult ALL (GMALL) [7], the regimen of the Dutch-Belgian Cooperative Trial Group for Hematology Oncology (HOVON) regimen [8], and the CODOX-M/IVAC regimen [9]. The complete response (CR) rates achieved with these regimens range between 72 and 89% [5–11].

Consensus on the optimal first line treatment for adult BL is still lacking. The low incidence of BL has thus far precluded direct comparison between treatment regimens in a randomized prospective clinical trial. Retrospective comparison of published clinical series is hampered by different patient selection criteria and changing histopathological definitions for aggressive B cell lymphoma including BL in successive WHO lymphoma classifications.

In the Netherlands, the LMB, BFM, HOVON, and CODOX-M/IVAC regimens are all in active use with treatment center preference based on historic and regional associations. Each of these regimens consists of a backbone of three to six courses of high-dose chemotherapy. The regimens differ in inclusion of maintenance therapy and autologous stem cell transplantation (SCT) as part of first line treatment, as well as agents used and dosages.

To support a rational choice for a standardized treatment of adult BL, we performed a retrospective observational analysis of real-world efficacy, toxicity, and costs of these four treatment protocols as used in daily clinical practice. In view of the evolving BL definitions over time, a central pathology assessment was included to guarantee meaningful comparisons.

## Patients and methods

### Patient selection and clinical data collection

All patients treated between 1995 and 2012 with any of the four treatment protocols under study in seven university medical centers and one non-academic tertiary referral hospital in the Netherlands were included in this study. Patients with prior first line treatment other than a maximum of three (R-) CHOP (-like) courses were excluded. Clinical data were collected from the hospital records using a standardized case report form. Adverse events were scored according to the Common Terminology Criteria for Adverse Events (CTCAE), Version 4.0. Specifically, infectious disease was defined as infections requiring intravenous antibiotic, antifungal, or antiviral intervention, or requiring radiologic or operative intervention; nephrotoxicity was defined as reduction of glomerular filtration rate to < 25% or creatinine increase > 3× baseline; hepatotoxicity was defined as elevation of transaminases > 5.0× upper normal level. Numbers of transfusions were extracted from the local blood bank databases. Treatment response was based on the original radiology reports (CT or PET-CT according to local practice). All treatment centers used the (revised) Cheson response criteria for response evaluation from 1999 onwards [12, 13]. Risk scores reported were the International Prognostic Index (IPI) score [12] and the Mead 2002 BL risk score [9].

### Central pathology assessment

Patients treated with the four treatment regimens under study were originally diagnosed with Burkitt lymphoma, small non-cleaved cell lymphoma, atypical Burkitt lymphoma, Burkitt-like lymphoma, B cell lymphoma with features intermediate between Burkitt lymphoma and diffuse large B cell lymphoma (BLU), Burkitt leukemia, mature B cell leukemia, or L3-leukemia according to the WHO lymphoma classification used at the time of diagnosis. Central pathology assessment was performed by two expert hematopathologists in two stages (D.d.J., P.M.K.). First, original pathology reports, including consult reports at the time of diagnosis, were reviewed. A case was accepted as BL if the following criteria were met: small- or medium-sized cells with monotonous morphology, proliferative index (MIB1) > 95%, and immunophenotype consistent with BL with BCL-2 staining negative or weak and CD10 and/or BCL-6 staining positive. A demonstrable MYC-translocation by cytogenetic testing or fluorescence in situ hybridization was considered supportive but not required for selection. Second, in those cases where the available pathology reports were insufficient or incomplete, complete formal review was performed, including additional BCL-2, CD10, and/or BCL-6 staining if not previously done. All cases not meeting the listed BL criteria were excluded from this study.

### Treatment regimens

Details on the treatment regimens included in this study are summarized in Table 1. All treatment regimens included rituximab from 2003 to 2004 onwards. Additional information can be found in the supplemental treatment regimen information.

**Table 1:** Chemotherapeutic drugs and drug dosages per treatment regimen

Treatment regimen^a^		LMB [6]	BFM [10]	HOVON [8]^b^	CODOX-M/IVAC [9]^c^	
Agents (no. courses × no. consecutive days)						
Alkylating agents	Cyclophosfamide	6800 (1 × 1 day, 2 × 3 days, and 2 × 2 days)	3000 (3 × 5 days)	7000 (3 × 1 day + 1 × 2 days)	3200 (2 × 5 days)	mg/m^2^
	Ifosfamide		8000 (2 × 5 days)		15,000 (2 × 5 days)	mg/m^2^
	Melphalan			140 (1 × 1 day)		mg/m^2^
	Carmustine			300 (1 × 1 day)		mg/m^2^
Antimetabolites	Cytarabine	25,500 (4 × 5 days continuous)	8600 (2 × 2 days, 2 × 1 day)	800 (1 × 4 days)	8000 (2 × 2 days)	mg/m^2^
	Methotrexate	9000 (4 × 1 day)	9000 (6 × 1 day)		13,440 (2 × 1 day)	mg/m^2^
Antitumor antibiotics	Adriamycine	240 (4 × 1 day)	200 (2 × 2 days)	280 (3 × 1 day,1 × 2 days)	80 (2 × 1 day)	mg/m^2^
Topoisomerase inhibitors	Etoposide	2500 (4 × 3 days)	1000 (2 × 2 days)	2800 (2 × 4 days)	600 (2 × 5 days)	mg/m^2^
	Mitoxantrone			30 (1 × 1 day)		mg/m^2^
	Teniposide		400 (2 × 2 days)			mg/m^2^
Mitotic inhibitors	Vincristine	12 (6 × 1 day)	8 (4 × 1 day)	6 (3 × 1 day)	8 (2 × 1 day)	mg
	Vindesine		10 (2 × 1 day)			mg/m^2^
Corticosteroids	Prednisolone	1740 (1 × 7 days, 4 × 5 days)	300 (1 × 5 days)	1250 (5 × 5 days)		mg/m^2^
	Dexamethasone		300 (6 × 5 days)			mg/m^2^
IT medication	Cytarabine	240 (8×)	160 (8×)		140 (2 × 1)	mg
	Methotrexate	90 (8×)	60 (8×)	75 (5×)	48 (4 × 1)	mg
	Dexamethason		32 (8×)			
	Hydroxycortison	160 (8×)				
Monoclonal antibodies	Rituximab^d^	1500 (4×)	3000 (8×)	1875 (5×)	2250 (6×)	mg/m^2^
Total number of courses		5 (low-int risk) 8 (high risk) ^e^	6	6	3 (low risk) 4 (high risk) ^e^	

^a^Doses given are for a patient < 40 years with a body surface area of 2 mg/m^2^ and a body weight of 75 kg, high risk BL without CNS involvement, and no dose reductions

^b^Preceded by 3× iR-CHOP from approximately 2005 onwards (included in calculations)

^c^No patients included in this study received dose-modified CODOX-M/IVAC

^d^Median number of rituximab gifts as administered per treatment regimen from 2003 to 2004 onwards

^e^Low-intermediate risk LMB, patients without bone marrow or CNS involvement; high risk LMB, all others patients; low risk CODOX-M/IVAC, BL score 0; and high risk CODOX-M/IVAC, BL score >0

### Statistical analyses

Baseline characteristics between the different treatment groups were compared using the *χ* ^2^ test or Fisher’s exact test for categorical data and the Kruskal-Wallis test for numerical data. Overall survival (OS) and progression-free survival (PFS) were calculated from start of the studied treatment regimen on an intention-to-treat basis and defined as the time to death from any cause (OS), and time to diagnosis of RD or relapse or death from any cause (whichever came first) (PFS). Survival of patients was censored at 5 years from the start of the studied treatment regimen or at the date of last contact, whichever came first. The Kaplan-Meier method was used to estimate OS and PFS, and to calculate 95% confidence intervals (CI). The log-rank test was used to compare survival between subgroups. Treatment-related mortality was calculated in a competing risks model in which lymphoma-related mortality was considered as a competing event. The cumulative incidence of relapse (CIR) was calculated in a competing risks model, considering non-relapse mortality as competing event. End of treatment was taken as starting time and patients with refractory disease were excluded from the analysis. Gray’s test was used to compare CIR between groups. Hazard ratios (HR) were calculated using univariate Cox regression models. Analyses were performed using SPSS, version 20, and R, version 3.3.0, with libraries “cmprsk” and “prodlim.” All reported *p* values are two-sided with a significance level of *α* = 0.05.

### Cost assessment

For each treatment regimen costs for medication use, erythrocyte and platelet transfusions, inpatients days and autologous graft collection (if applicable) were calculated, for an “average” patient based on the following assumptions: < 40 old, body surface area of 2 m^2^, weight of 75 kg, high risk BL without CNS involvement, no dose reductions, administration of the median number of rituximab gifts and the mean number of blood product transfusions, and hospital admission for the mean number of days as reported for that treatment regimen. Costs of supportive medication and outpatient evaluations were not taken into account but were assumed to be comparable between all regimens. All costs are reported in Euro and indexed to the year 2015 using the Dutch consumer price index as published on the CBS Statistics Netherlands website [14]. Sources used were the knowledge database of the Royal Dutch Pharmacists Association (z-index) [15] per December 2015 (costs for carmustine and vindesine were obtained from the Leiden University Medical Center Pharmacy), the Dutch guideline for economic evaluations [16], and previous published studies [17–19].

## Results

### Patient characteristics and chemotherapy regimens

**Fig. 1:**
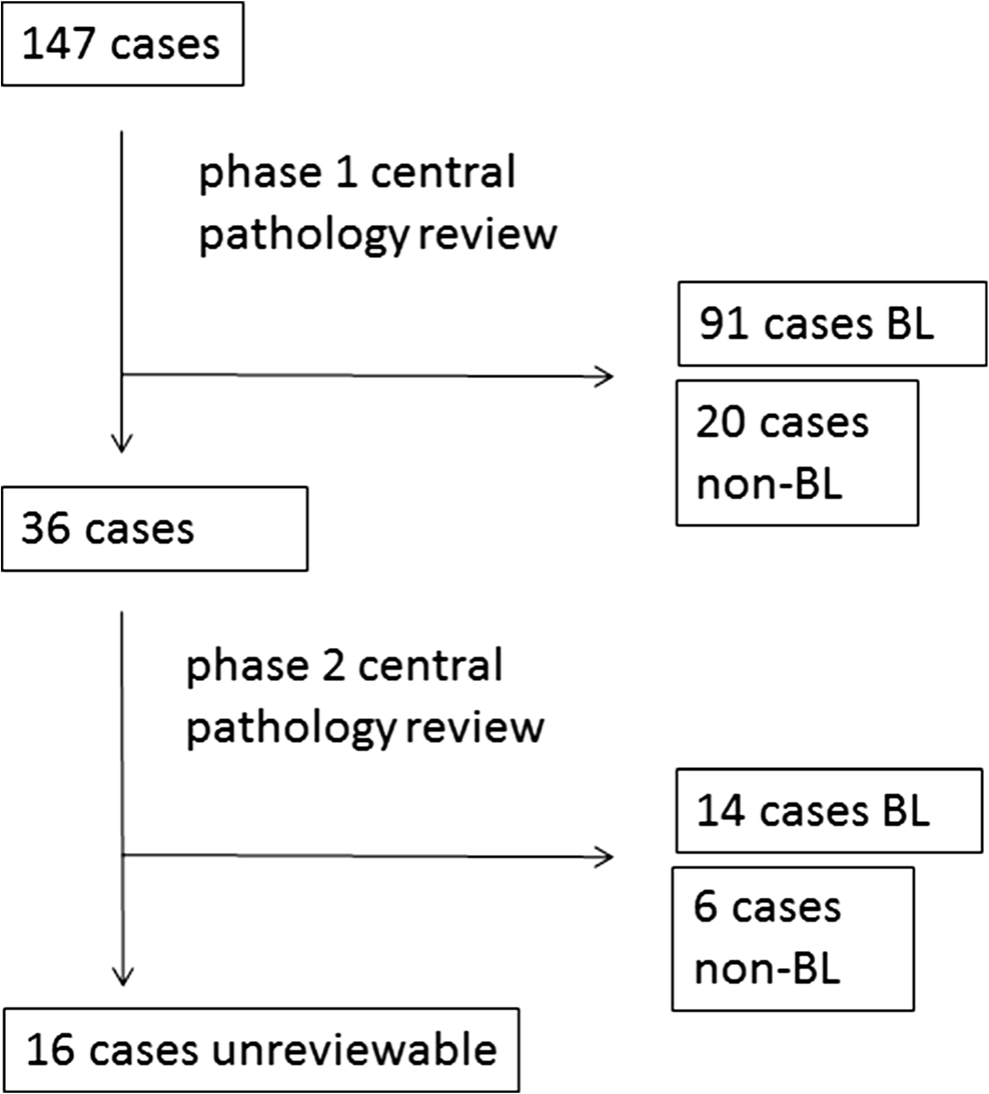
Central pathology assessment flow chart

A total of 147 adolescent and adult patients (14–74 years) treated for BL with the LMB, the BFM, the HOVON, or the CODOX-M/IVAC regimen between 1995 and 2012 were identified (Fig. 1). Of these, 91 cases fulfilled the criteria for BL in the first phase of the pathology assessment and 14 additional cases after complete assessment resulting in a total of 105 confirmed BL cases. Twenty-six cases were rejected as non-BL. Sixteen cases were considered unreviewable.

### Patient characteristics and chemotherapy regimens

Baseline patient characteristics are listed in Table 2. Thirty-six patients were treated with the LMB regimen, 19 with the BFM regimen, 29 with the HOVON regimen, and 21 with the CODOX-M/IVAC regimen. Of the LMB patients, 11 received the low-intermediate risk schedule (31%). Of the CODOX-M/IVAC patients, five received the low risk schedule consisting of three CODOX-M courses (24%). Rituximab was included in the treatment regimen of 64% (LMB) to 100% (CODOX-M/IVAC) of patients. The treatment groups were similar in most characteristics, with the following exceptions. The HOVON group contained fewer patients with central nervous system (CNS) involvement (*p* = 0.065), no patients with leukemic BL (n.s.), and fewer patients with high WHO scores (*p* = 0.002), resulting in slightly better overall IPI scores (*p* = 0.031). The BFM group contained fewer patients with ≥ 2 extranodal sites (*p* = 0.037). Lastly, the LMB and HOVON groups had fewer patients treated with rituximab (*p* = 0.003) than the BFM and CODOX-M/IVAC groups. Since the latter regimens were introduced more recently in the Netherlands, they had a higher percentage of patients included after 2003–2004.

**Table 2:** Patient characteristics per treatment regimen

Treatment regimen	LMB	BFM	HOVON^a^	CODOX-M/IVAC	All	*P* value
*N*	36	19	29	21	105	
Age median (range)	35 (14–74)	40 (19–57)	39 (15–57)	39 (17–62)	39 (14–74)	0.784
< 40 years	22 (61%)	9 (47%)	15 (52%)	11 (52%)	57 (54%)	
≥ 40 years	14 (39%)	10 (53%)	14 (48%)	10 (48%)	46 (46%)	
Sex						0.098
Male	27 (75%)	9 (47%)	23 (79%)	14 (67%)	73 (70%)	
Female	9 (25%)	10 (53%)	6 (21%)	7 (33%)	32 (30%)	
Ann Arbor stage						0.695
I–II	9 (25%)	3 (16%)	9 (31%)	5 (24%)	26 (25%)	
III–IV	27 (75%)	16 (84%)	20 (69%)	16 (76%)	79 (75%)	
Extranodal involvement						
Bone marrow	12 (34%)	7 (37%)	7 (24%)	9 (43%)	35 (34%)	0.559
Central nervous system	8 (22%)	5 (26%)	3 (10%)	9 (43%)	25 (24%)	0.065
Gastrointestinal tract	13 (36%)	10 (53%)	14 (48%)	6 (29%)	43 (41%)	0.335
≥ 2 sites	19 (54%)	2 (14%)	8 (29%)	9 (43%)	38 (39%)	0.037
LDH > upper normal level	28 (80%)	14 (78%)	17 (63%)	16 (76%)	75 (74%)	0.464
WHO performance score^b^						0.002
0–1	20 (56%)	6 (55%)	22 (92%)	19 (73%)	67 (73%)	
> 1	16 (44%)	5 (45%)	2 (8%)	2 (27%)	25 (27%)	
Bulky disease ≥ 10 cm	9 (27%)	2 (11%)	6 (21%)	7 (33%)	24 (24%)	0.371
Peripheral blood blasts ≥ 30%	1 (3%)	2 (2%)	0	2 (10%)	5 (5%)	0.224
IPI-score ^c^						0.031
0–2	11 (31%)	6 (38%)	17 (65%)	12 (57%)	46 (47%)	
3–5	25 (69%)	10 (62%)	9 (35%)	9 (43%)	53 (53%)	
BL-risk score^c^						0.874
Low	4 (11%)	1 (6%)	3 (12%)	3 (14%)	11 (11%)	
High	32 (89%)	16 (94%)	23 (89%)	18 (86%)	89 (89%)	
HIV positivity	3 (9%)	1 (6%)	6 (21%)	3 (16%)	13 (13%)	0.384
Rituximab in regimen	23 (64%)	18 (95%)	23 (79%)	21 (100%)	85 (81%)	0.003
Lymphoma treatment prior to initiation of studied regimen^d^	3 (8%)	5 (26%)	3 (10%)	1 (5%)	12 (11%)	0.141

*LDH* lactate dehydrogenase, *HIV* human immunodeficiency virus

^a^Seven of these patients were included in the original prospective HOVON 27 study [8]

^b^WHO performance score data missing for 13 patients, resulting in incalculable IPI-scores for 6 patients

^c^International Prognostic Index (IPI) score: age > 60 years, Ann Arbor stage III/IV disease, elevated serum lactate dehydrogenase (LDH), WHO performance score > 1, > 1 extranodal site (1 point for each) [20]; BL risk score: Ann Arbor stage III/IV disease, elevated serum LDH, WHO performance score > 1, and bulky disease (1 point for each) [9]

^d^Maximum of 3 (R-) CHOP (-like) courses

Seventy-five patients (71%) completed the planned treatment regimen without treatment modifications. Five patients switched to palliative therapy due to progressive disease (two LMB, one BFM, and two HOVON). Twenty patients switched to more intensive therapy or received additional chemotherapy courses due to insufficient response or heightened risk as perceived by the treating physician (11 LMB, 2 BFM, 6 HOVON, and 1 CODOX-M/IVAC). Three patients received fewer courses than planned or switched to less intensive therapies due to toxicity or comorbidity (two LMB, one HOVON). Two patients died early during treatment (one LMB, one CODOX-M/IVAC). For the different treatment regimens, the percentage of patients completing planned treatment modifications was respectively 56% for LMB, 84% for BFM, 69% for HOVON, and 91% for CODOX-M/IVAC (*p* = 0.020).

### Response rates and survival

Clinical outcomes related to chemotherapy regimens are shown in Table 3. Median follow-up of all patients was 47 months (range 4–172 months). Duration of follow-up was variable between treatment groups (*p* = 0.002) due to CODOX-M/IVAC having been introduced more recently (maximum follow-up duration 75 months). To minimize potential effects of late non-relapse mortality in the other three treatment groups (maximum follow-up duration 147–172 months), survival was censored at 5 years.

**Table 3:** Outcomes per treatment regimen

Treatment regimen	LMB	BFM	HOVON	CODOX-M/IVAC	All	*P* value
*N*	36	19	29	21	105	
Response rates						
Complete response	22 (65%)	10 (53%)	20 (69%)	15 (75%)	67 (66%)	0.501
Partial response	5 (15%)	4 (21%)	3 (10%)	2 (10%)	14 (14%)	0.705
Refractory disease	7 (21%)	5 (26%)	6 (21%)	3 (15%)	21 (21%)	0.858
Not evaluable	2 (6%)	0	0	1 (5%)	3 (3%)	
Relapse rates						
Relapse (at 1 year after end of treatment, corrected for competing events)	7% (95% CI 0–17%)	0% (95% CI 0–0%)	9% (95% CI 0–21%)	12% (95% CI 0–28%)	7% (95% CI 2–13%)	0.612
5-year survival rates						
Progression-free survival	67% (95% CI 53–80%)	74% (95% CI 57–91%)	68% (95% CI 53–83%)	71% (95% CI 54–88%)	69% (95% CI 60–78%)	0.966
Overall survival	66% (95% CI 53–80%)	74% (95% CI 54–93%)	71% (95% CI 54–88%)	70% (95% CI 50–90%)	69% (95% CI 60–78%)	0.981

Of the 67 patients who achieved CR (Table 3), four patients relapsed (6%) and eventually died of disease progression. Two of these patients had been treated with the LMB regimen and two with the HOVON regimen. One patient in CR received intensification therapy including an allogeneic SCT directly following the treatment regimen because of extensive CNS involvement at presentation but died due to treatment-related complications. All other patients with initial complete responses were alive at the end of follow-up (93%).

**Fig. 2:**
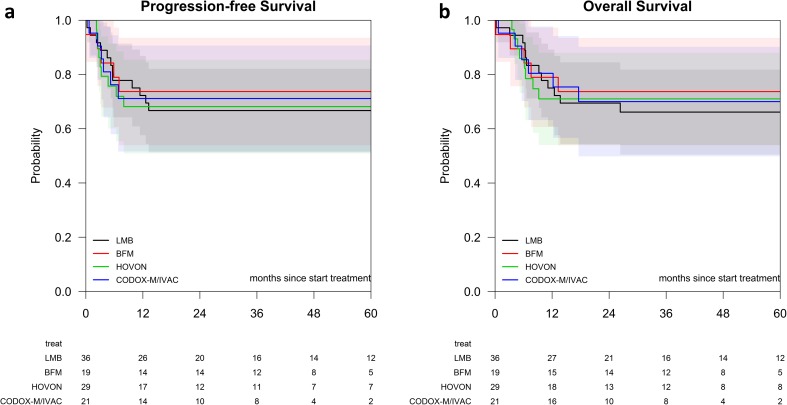
**a** Progression-free survival of BL patients treated with the LMB, BFM, HOVON, or CODOX-M/IVAC regimens. **b** Overall survival of BL patients treated with the LMB, BFM, HOVON, or CODOX-M/IVAC regimens. LMB, black line; BFM, red line; HOVON, green line; and CODOX-M/IVAC, blue line

There were no significant differences between the treatment regimens with respect to progression-free survival and overall survival, response rates, and relapse rates (Fig. 2 and Table 3). Because advanced patient age has been associated with poorer outcome in BL and the different treatment regiments might impact older patients differently, we stratified for patient age < 40 years versus patient age ≥ 40 years. No significantly different survival rates emerged between the treatment regimens for the different age groups (*p* = 0.991 for 5-year OS of patients < 40 years, *p* = 0.845 for 5-year OS of patients ≥ 40 years). Likewise, no different survival rates were detected following stratification for low (0–2) versus high (3–5) IPI scores (*p* = 0.885 for 5-year OS of patients with low IPI scores, *p* = 0.841 for 5-year OS of patients with high IPI scores). We could make no calculations for low versus high BL scores due to insufficient events in the low BL score group. Prognostic factors and survival rates for the various risk groups are listed in the Supplementary Material.

### Toxicity and treatment-related mortality

Transfusion requirements and CTCAE grade III–IV infectious disease, nephrotoxicity, or hepatotoxicity occurred in all four treatment groups. The affected percentage of patients was as follows for the LMB, BFM, HOVON, and CODOX-M/IVAC regimens, respectively: transfusion requirement 97, 95, 88, and 100% (*p* = 0.386); infectious disease 71, 63, 52, and 86% (*p* = 0.095); nephrotoxicity 11, 5.3, 4, and 7% (*p* = 0.672); hepatotoxicity 29, 26, 4, and 53% (*p* = 0.004). No significant difference in transfusion requirement or the occurrence of these toxicities was detected between patients < 40 years and patients ≥ 40 years.

Treatment-related deaths were rare: two patients died due to sepsis during the first course of chemotherapy) (one CODOX-M/IVAC, one LMB), five patients died from treatment-related complications from intensification therapy, including the three patients who received an allogeneic SCT. Two patients died during follow-up from unknown causes. Treatment-related mortality corrected for competing events was 5% (95% CI, 1–9%) at 1 year and 6% (95% CI, 1–10%) at 2 years after start of treatment.

### Treatment of partial response, refractory disease, and disease relapse

Treatment strategies for partial response, refractory disease, and disease relapse were highly variable and dependent on patient condition, extent of disease, and earlier treatment. Of the 14 patients with PR, 9 patients received additional therapy. Four of these patients received local radiation therapy only, none of whom relapsed. The other five patients received additional chemotherapy, followed in three cases by autologous SCT. All of these patients achieved prolonged remissions. Of the untreated patients, two relapsed and one died of unknown causes. The other two patients remained in remission. Overall, 5-year OS of PR patients was 79% (median follow up 51 months, range 18–172 months) as opposed to 92% 5-year OS of CR patients. Of the 22 patients with RD, 21 died due to disease progression, one patient achieved remission after an intensive chemotherapy schedule.

All patients who relapsed eventually died, either due to disease progression or to complications of therapy. Median duration of disease-free survival in patients who relapsed was 3.5 months to first relapse. All relapses but one occurred within the first 9 months. In total, 23 of the 31 patients that were no longer alive at the end of follow-up died due to progressive disease.

### Duration and costs of treatment

For all patients who completed high-risk protocol treatment without treatment schedule modifications, we calculated total duration of treatment and number of inpatient days as well as treatment costs (Table 4). The CODOX-M/IVAC regimen had the overall shortest duration of treatment (95 days versus 149–231 days for the other regimens). The HOVON regimen had the lowest number of inpatient days (63 days versus 93–134 days for the other regimens). The low risk variants of CODOX-M/IVAC and LMB (not shown in table) had median treatment duration of 78 days (range 68–85) and 102 days (range 90–159) and median inpatient treatment of 58 days (range 45–62) and 51 days (range 38–66), respectively.

**Table 4:** Duration and cost of treatment per treatment regimen

Treatment regimen	LMB^a^	BFM	HOVON^b^	CODOX-M/IVAC	*P* value
*N*	13	16	18	15	
Median number of treatment days (range)					
Duration of treatment according to protocol	217	168	105	84	
Observed duration of treatment	231 (193–319)	171 (146–226)	149 (121–215)	95 (80–155)	< 0.001
Planned number of inpatient days according to protocol	129	147	66	84	
Observed number of inpatient days	102 (70–148)	134 (100–169)	63 (53–109)	93 (75–130)	< 0.001
Treatment costs in €					
Medication costs excluding rituximab	5940	7702	11,272	9323	
Rituximab	7282	14,564	9103	10,923	
Blood product transfusions	14,964	5349	4838	16,204	
Diagnostic procedures	18,627	27,941	13,971	18,627	
Inpatient days	64,063	86,320	46,959	55,991	
Autologous graft mobilization and harvest^c^	–	–	5251	–	
Total costs in €	110,876	141,877	91,394	111,068	

^a^Data shown for high risk protocol

^b^Including 3× RiCHOP

^c^Costs for daycare, medication, laboratory activities, and apheresis procedure related to mobilization/harvesting of autologous graft

For treatment costs, the number of inpatient days and the number of rituximab gifts were the most important determinants. The number of rituximab gifts was not defined in the original treatment protocols. In practice, most treatment centers chose to administer 1–2 rituximab gifts per chemotherapy course in varying schedules. In the treatment regimens evaluated in this study, the number of rituximab gifts varied from four (LMB regimen) to eight (BFM regimen). The HOVON regimen was associated with the lowest total costs mainly due to the lowest number of inpatient days (91,394 €). The LMB regimen was associated with the lowest drug costs mainly due to the lowest number of rituximab administrations (13,222 € drug cost). The BFM protocol carried the highest costs as it comprised both the highest number of inpatient days as well as the highest cumulative rituximab dose (141,877 €).

## Discussion

In the absence of a standard first line treatment of adult BL, we studied “real-world” efficacy, toxicity, and costs of four BL regimens frequently used in the Netherlands: LMB, BFM, HOVON, and CODOX-M/IVAC. Our aim was to support an evidence-based choice for a first line BL treatment regimen. Central pathology assessment was performed to ensure inclusion only of BL cases selected according to 2008 WHO classification criteria. These criteria remain essentially unchanged in the upcoming WHO 2016 classification [21], so that our study population remains a good approximation also of the WHO 2016 BL population.

Having validated the diagnosis, we found patient selection in the four treatment groups to be mostly similar in terms of age, known risk factors, and composite indices. The HOVON treatment group contained relatively fewer high-risk patients because patients with CNS disease and leukemic BL were excluded from the HOVON27 trial, which included patients up to 2003 [8], and were also not routinely treated with this regimen in the years that followed. In the HOVON and LMB treatment groups, fewer patients received rituximab as a higher percentage of patients started treatment prior to 2003–2004. Despite these differences, we found PFS and OS rates to be comparable between the four treatment regimens. Addition of rituximab did not significantly affect OS but did show a possible trend toward improved survival especially in the older patient groups (Supplemental Material on prognostic factors). Our data do not allow us to draw conclusions on the optimal number of rituximab gifts.

Next to efficacy, the toxicity spectrum could serve as an important parameter for optimal treatment choice. Overall, the treatment regimens seem to be comparably safe. Only hepatotoxicity was significantly different between the treatment regimens and highest for the CODOX-M/IVAC regimen. The relevance of this finding is unclear as dose adjustment for hepatotoxicity was reported for only one patient during first line treatment (LMB regimen). Since our analysis was limited to toxicities that could be quantifiably extracted from patient records, mucositis was not evaluated despite being a frequent cause of morbidity in patients undergoing intensive chemotherapy. In prospective studies (Table 5), incidence of mucositis was reported as 12–14% for LMB [6], 29% for BFM [10], 39% for HOVON [8], and 50% for CODOX-M/IVAC [9]. While these data imply that the CODOX-M/IVAC regimen may be more strongly associated with mucositis than the other treatment regimens, this did not reflect in a diminished percentage of patients completing treatment.

**Table 5:** Summary of literature on studied treatment regimens

Study	Protocol	*N* (enrollment)	Median age	Rituximab included	CR (%)	OS	NRM (%, years)
Soussain et al. [5]	LMB retrospective	65 (1984–1991)	26	No	89	74% (3 years)	12 (1)
Diviné et al. [6]	LMB prospective	72 (1996–2001)	33	No	72	70% (2 years)	4 (1)
Choi et al. [22]	LMB retrospective	38 (1998–2008)	47	No	74	68% (1 year)	11 (1)
Hoelzer et al. [7]	BFM prospective	35 L3-ALL only (1991-nr)	36	No	74	51% (4 years)	6 (4)
Tauro et al. [23]	BFM retrospective	46 (nr-nr)	32	No	87	80% (2 years)	Nr
Pohlen et al. [24]	BFM retrospective	28	Nr		84	Nr	7 (5)
Intermesoli et al. [25]	BFM retrospective	105 (2002–2010)	47	Yes	79	67% (3 years)	18 (3)
Hoelzer [10]	BFM prospective	263 (2002–2011)	42	Yes	88	70% (5 years)	5 (5)
van Imhoff et al. [8]	HOVON27 prospective	27 (1994–2003)	36	No	81	81% (5 years)	0 (5)
Mead et al. [9]	CODOX-M/IVAC prospective	52 (1995–1999)	35	No	89	73% (2 years)	13 (2)
Lacasce et al. [26]	dmCODOX -M/IVAC prospective	14 (nr)	47	No	86	64% (2 years)	0 (2)
Mead et al. [11]	dmCODOX-M/IVAC prospective	53 (2002–2005)	37	No	74	67% (2 years)	8 (2)
Maruyama et al. [27]	CODOX-M/IVAC retrospective	15	39		87	87% (5 years)	0 (5)
Barnes et al. [28]	dmCODOX-M/IVAC retrospective	80 (1992–2009)	46	No (40), yes (40)	88	71% (3 years)	8 (3)
Wästerlid et al. [29]	BFM, CODOX-M/IVAC	71, 32	40, 42		Nr, nr	82, 69%	Nr, nr

*CR* complete response following protocol treatment, *OS* overall survival, *NRM* non-relapse mortality, *Nr* not reported

As the final important parameter to guide medical decision-making, we evaluated the actual costs of the various treatment regimens and the treatment durations. Treatment duration is an important determinant from a comprehensive health economics point of view as it affects the time period a patient is impaired at work and at home. Dominant drivers of treatment cost were length of in-hospital stay (€ 640 per day) and the cumulative rituximab dose (€ 1821 per gift) for medication costs. The HOVON regimen was associated with the shortest in-hospital stay and the lowest medication costs. CODOX-M/IVAC was associated with the shortest total treatment duration and the second shortest duration of in-hospital stay and medication costs. Medication costs for all regimens are likely to decrease in the future as biosimilars of rituximab become available.

With efficacy and safety comparable between the four treatment regimens and health economics favoring the HOVON and CODOX-M/IVAC regimens, we weighed the advantages and disadvantages of these two regimens with regard to the choice for a standard first line BL therapy. Although the HOVON treatment group contained less high risk patients, the CODOX-M/IVAC regimen performed equally well and was completed by a significantly higher percentage of patients without significant treatment modifications. The CODOX-M/IVAC regimen has the further advantage of a low risk protocol variant with reduced doses of alkylating agents. Since these drugs are most commonly associated with chemotherapy-induced infertility, this is of relevance for the low risk population that mostly comprises young patients [30]. Based on these considerations, the CODOX-M/IVAC regimen seems the most rational choice for a standard first line BL therapy.

There are limitations to our study due to its retrospective observational nature. One important aspect pertains to possible treatment center-related differences that might affect outcome. The LMB-, HOVON-, and CODOX-M/IVAC regimens were each practiced in two or three hospitals lessening the impact of center-specific policies, but the BFM regimen was practiced in one center only. The survival rates we found are nevertheless comparable to those published in prospective studies of the individual treatment regimens and a population-based study comparing BFM, CODOX/M-IVAC, hyper-CVAD, and CHOP/CHOEP regimens (Table 5), externally validating our results. Another limitation results from the changing standards for response evaluation from 1995 to 2012, initially involving CT and later PET-CT imaging. In our study, two patients with PR did not receive intensification therapy but nevertheless remained in remission and may in fact have had a (unconfirmed) CR. Unfortunately, we could not perform a central radiology review, but even with the current standard of care, response evaluation in BL is known to be difficult. In a study of 27 BL patients with post-treatment PET-CT, positive predictive value was only 20% (negative predictive value was 100%) [31]. A study in pediatric Burkitt patients likewise showed a tendency for false positives due to acute inflammation and tumor necrosis [32]. In our study, in one PR patient, an extirpation was performed of a single residual mass that remained PET-positive despite intensification therapy, revealing an absence of vital BL tissue. This was not, however, routinely done. We believe in selected cases it may be prudent to strive for pathological confirmation of positive PET/CT results following end of treatment to avoid unnecessary intensification.

An issue highlighted by our study, is the current lack of effective treatment for refractory or relapsed patients. Of the 22 patients in our study that did not have a complete or partial response, 21 died, as well as all patients that relapsed. Escalation to autologous SCT is the best documented treatment option [33, 34]. A 2013 study from the Center for International Blood and Marrow Transplant Research reported on 241 patients receiving an autologous or allogeneic SCT for BL in second or subsequent CR [34]. The 5-year OS was shown to be 31% following autologous SCT and 20% following allogeneic SCT. Patients not in CR at the time of transplant had 5-year OS of 22 and 12%, respectively. In our patient cohort, three patients received allogeneic SCT as intensification therapy or following relapse but all patients died due to treatment-related complications. Autologous SCT did seem to be effective, but only for patients with chemosensitive disease and would be our treatment of choice for patients with PR or relapsed BL. Patients with refractory BL may be better served with novel therapies targeting contributing pathways in an experimental trial setting.

Recently, DA-EPOCH-R was proposed as a novel, highly promising first line treatment regimen [35]. With this dose-adjusted low intensity regimen, 87% freedom of disease progression was reported in 77 patients at a median follow-up of 25 months with relatively limited side effects [36]. Since this treatment can be administered in the outpatient setting, it is attractive from a health economics point of view. Based on the advantages with respect to cost and treatment duration, our data have led to the choice of the CODOX-M/IVAC regimen as the standard arm in the multinational randomized prospective HOVON127 trial that is designed to assess the possible superiority of DA-EPOCH-R (EU Clinical Trials register, EudraCT 2013-004394-27). Also, our data have led at least two treatment centers in the Netherlands to adopt CODOX-M/IVAC as BL treatment of choice.

In summary, our study demonstrates high cure rates for BL in a real-world setting. Given the lack of major differences in outcome and toxicity, health economic aspects may guide the choice of treatment.

## Electronic supplementary material

ESM 1 (DOCX 145 kb).

ESM 2 (DOCX 5443 kb).
