# Supplementary material for: Treatment of sporadic Burkitt lymphoma in adults, a retrospective comparison of four treatment regimens
Source: Ann Hematol. 2017 Dec 6;97(2):255–66. doi: 10.1007/s00277-017-3167-7 (PMC5754407; doi:10.1007/s00277-017-3167-7)
Supplement: Supplementary file 1 — (DOCX 145 kb). [file 277_2017_3167_MOESM1_ESM.docx]

**
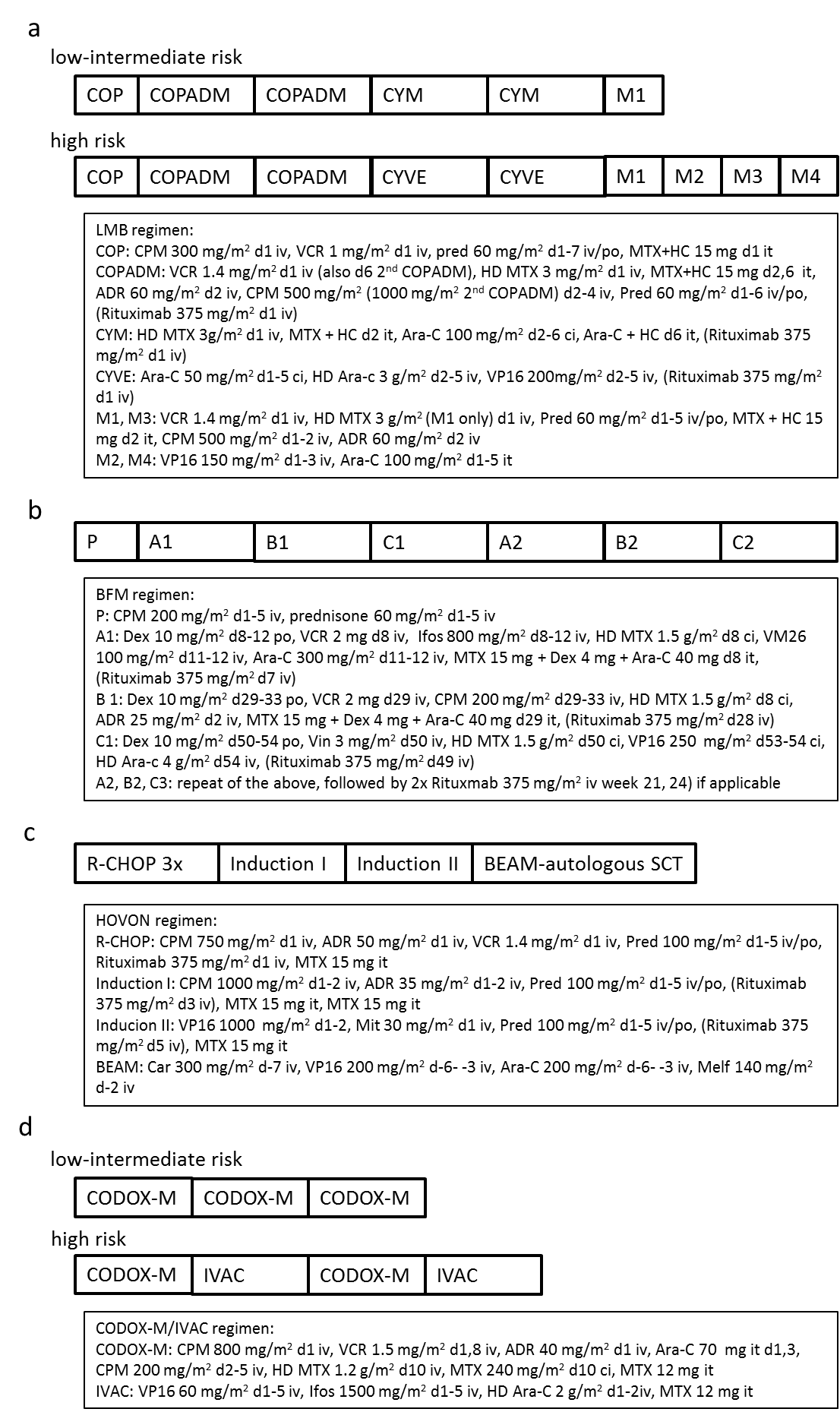
Supplemental treatment regimen information**

**Supplemental Fig. 1:** (a) LMB regimen [1]. (b) BFM regimen [2]. (c) HOVON regimen [3]. (d) CODOX-M/IVAC regimen [4]. Ara-C: cytarabine; ADR: adriamycin/doxorubicin; Car: carmustine; CPM: cyclophosphamide; Dex: dexamethasone; HC: hydrocortisone; Ifos: ifosfamide; Melf: melfalan; Mit: mitoxantrone; MTX: methotrexate; Pred: prednisone; VCR: vincristine; Vin; vindesine; VM26: tenoposide; VP16: etoposide; ci: continuous infusion; it: intrathecal; iv: intravenous; po: oral; sc: subcutaneous; HD: high dose, d: day from start cycle. Cycle names and schedules are listed as in the original reference. For information on regimen-specific risk stratification, leucovorin and granulocyte- colony stimulating factor rescue schedules, dose adaptations for age and central nervous system involvement, please refer to the original reference.

**Supplemental treatment regimen information References**

1. Divine M, Casassus P, Koscielny S, Bosq J, Sebban C, Le Maignan C, Stamattoulas A, Dupriez B, Raphael M, Pico JL, Ribrag V, Goelams G (2005) Burkitt lymphoma in adults: a prospective study of 72 patients treated with an adapted pediatric LMB protocol. Ann Oncol 16 (12):1928-1935. doi:DOI 10.1093/annonc/mdi403

2. Hoelzer D, Walewski J, Dohner H, Viardot A, Hiddemann W, Spiekermann K, Serve H, Duhrsen U, Huttmann A, Thiel E, Dengler J, Kneba M, Schaich M, Schmidt-Wolf IG, Beck J, Hertenstein B, Reichle A, Domanska-Czyz K, Fietkau R, Horst HA, Rieder H, Schwartz S, Burmeister T, Gokbuget N, German Multicenter Study Group for Adult Acute Lymphoblastic L (2014) Improved outcome of adult Burkitt lymphoma/leukemia with rituximab and chemotherapy: report of a large prospective multicenter trial. Blood 124 (26):3870-3879. doi:10.1182/blood-2014-03-563627

3. van Imhoff GW, van der Holt B, MacKenzie MA, Ossenkoppele GJ, Wijermans PW, Kramer MHH, van't Veer MB, Schouten HC, Kooy MV, van Oers MHJ, Raemaekers JMM, Sonneveld P, Meulendijks LAMH, Kluin PM, Kluin-Nelemans HC, Verdonck LF (2005) Short intensive sequential therapy followed by autologous stem cell transplantation in adult Burkitt, Burkitt-like and lymphoblastic lymphoma. Leukemia 19 (6):945-952. doi:DOI 10.1038/sj.leu.2403733

4. Mead GM, Sydes MR, Walewski J, Grigg A, Hatton CS, Norbert P, Guarnaccia C, Lewis MS, McKendrick J, Stenning SP, Wright D, Collaborators UL (2002) An international evaluation of CODOX-M and CODOX-M alternating with IVAC in adult Burkitt's lymphoma: results of United Kingdom Lymphoma Group LY06 study. Ann Oncol 13 (8):1264-1274. doi:DOI 10.1093/annonc/mdf253
