# Supplementary material for: Treatment of sporadic Burkitt lymphoma in adults, a retrospective comparison of four treatment regimens
Source: Ann Hematol. 2017 Dec 6;97(2):255–66. doi: 10.1007/s00277-017-3167-7 (PMC5754407; doi:10.1007/s00277-017-3167-7)
Supplement: Supplementary file 2 — (DOCX 5443 kb). [file 277_2017_3167_MOESM2_ESM.docx]

**Supplemental Data**

*Prognostic factors*

Hazard ratios at univariate levels for a number of potentially significant prognostic factors are listed in supplementary table 1. Age was the only factor that showed statistical significance in this patient population. Significance for age remained when stratified into two age groups: < 40 years and ≥ 40 years at diagnosis. Supplemental figure 2 a and b show OS for each of the age categories per treatment regimen. It should be noted that older patients generally presented with more advanced disease than the younger patients, as evidenced by higher IPI scores (p=0.032) and BL scores (p=0.038). The decrease in OS for patients ≥ 40 years was almost exclusively due to lymphoma-related death. IPI score < 2 and ≥ 2, but not BL score, was found to significantly predict OS. Supplemental figure 2 c and d show OS for low and high IPI scores per treatment regimen. 5-year OS per IPI score was 75% for 0 points, 90% for 1 point, 79% for 2 points, 57% for 3 points, and 62% for 4-5 points.

In order to study whether improved supportive care over the years might have improved survival among patients with BL, we included the year of diagnosis as a variable. No significant effect was found. Also, we evaluated OS for the various treatment groups corrected for WHO score because the WHO performance score was significantly lower for the HOVON patient group than for the other groups. This did not notably affect the HR. The addition of rituximab to the treatment regimen did not significantly affect OS, but we did notice a trend toward significance for the age group ≥ 40 years (OS 33% versus OS 67%, p=0.088) as opposed to little improvement for the age group < 40 years (OS 73% versus 80%, p=0.545).

Several studies have evaluated prognostic factors in BL. Advanced age using various cut-off points [1-6], performance score [2,7,5], race [1,3], gender [6], LDH elevation [2,4], Ann Arbor stage [1], and extranodal involvement [4,8,5], have all been found relevant, but studies vary considerably in which factors are found to be statistically significant. In our study, patient age was the only prognostic factor that reached statistical significance. The impact of age is likely multifactorial and may reflect treatment toxicity and a higher incidence of complex karyotype abnormalities impacting response to therapy. Cause of death in our study in patients ≥ 40 years was mainly lymphoma related, but a role of dose reductions or delays due to toxicity cannot be excluded. We chose to stratify patients in age cohorts of < 40 versus ≥ 40 years because 40 years is the cut off reported in three large population based studies evaluating 284, 2284, and 3691 BL patients [9,3,1].

Currently, there is no consensus as to the optimal prognostic score for BL. The IPI-score that is commonly used for BL risk classification has been designed and validated for aggressive lymphoma treated with CHOP-like chemotherapy but not for BL. The IPI-score assigns one point to patient age > 60 years, WHO performance score > 1, elevated serum LDH, Ann Arbor stage III or IV, and ≥ 1 extranodal site [10]. For our patient population, when we adjusted the IPI score to assign one point for patient age ≥ 40 instead of 60, the IPI score became a far more accurate predictor of OS, resulting in a 5 year OS of 88%, 82%, 79%, 65%, and 56% for patients with scores of 0, 1, 2, 3, 4-5 respectively. It should be noted that our patient population only comprises patients considered fit enough to undergo dose-intensive chemotherapy so this score should of course be validated in a general BL population that also includes elderly patients. A simpler score was recently suggested by Wästerlid et al. in a study on prognostic factors in 156 patients collected from the Swedish Lymphoma Registry [2]. He suggested a three tier prognostic index consisting of age > 40 years (1 point), LDH > upper normal level (1 point), WHO performance score > 1 (1 point). This score resulted in 5 year OS of 91%, 67%, 67% and 55% for patients with scores of 0, 1, 2, and 3 points respectively in our patient population. Recently, in the largest study on prognostic factors in BL to date, Castillo et al. evaluated 2284 patients with BL from the Surveillance, Epidemiology and End Results (SEER database). In this study, older age, black race, and advanced stage were found to be significant [9]. Unfortunately, data on WHO performance score, LDH elevation, and extranodal involvement where not evaluated. Based on his findings, Castillo devised a prognostic score assigning 1 point for age > 40-59 or black race, 2 points for age 60-79 or Ann Arbor stage III-IV disease, and 4 points for age ≥ 80. We could not validate this score in our own patient population as our standardized case report forms did not include data on race.

Risk stratification for the purpose of assigning patients to a low risk or high risk treatment schedule such as required for the LMB and CODOX-M/IVAC regimens, tends to be based on tumor markers only. For the LMB regimen, all patients with bone marrow and/or CNS involvement were considered high risk, the remainder were considered low-intermediate risk [4]. The original protocol also contained a low risk group consisting of patients with completely resected stage I and abdominal stage II disease but no patients in our study received the associated low risk protocol. For the CODOX-M/IVAC regimen, Mead et al. in 2002 defined patients as low risk if they had none of the following: serum LDH elevation, WHO performance score > 1, Ann Arbor stage III-IV, bulky disease, referred to in this article as the BL score [11]. A 2014 review article by Jacobson et al. on the treatment of BL using CODOX-M/IVAC defined low risk as a single site of disease < 10 cm and normal LDH [12]. The treatment centers participating in our study used varying risk stratifications over time. As such we cannot make recommendations with regards to the best risk stratification strategy. Both the BL score and the low risk definition by Jacobson et al. seem acceptable options.

**Supplemental Data References**

1. Castillo JJ, Winer ES, Olszewski AJ (2013) Population-based prognostic factors for survival in patients with Burkitt lymphoma: an analysis from the Surveillance, Epidemiology, and End Results database. Cancer 119 (20):3672-3679. doi:10.1002/cncr.28264

2. Wasterlid T, Jonsson B, Hagberg H, Jerkeman M (2011) Population based study of prognostic factors and treatment in adult Burkitt lymphoma: a Swedish Lymphoma Registry study. Leuk Lymphoma 52 (11):2090-2096. doi:10.3109/10428194.2011.593274

3. Costa LJ, Xavier AC, Wahlquist AE, Hill EG (2013) Trends in survival of patients with Burkitt lymphoma/leukemia in the USA: an analysis of 3691 cases. Blood 121 (24):4861-4866. doi:10.1182/blood-2012-12-475558

4. Divine M, Casassus P, Koscielny S, Bosq J, Sebban C, Le Maignan C, Stamattoulas A, Dupriez B, Raphael M, Pico JL, Ribrag V, Goelams G (2005) Burkitt lymphoma in adults: a prospective study of 72 patients treated with an adapted pediatric LMB protocol. Ann Oncol 16 (12):1928-1935. doi:DOI 10.1093/annonc/mdi403

5. Intermesoli T, Rambaldi A, Rossi G, Delaini F, Romani C, Pogliani EM, Pagani C, Angelucci E, Terruzzi E, Levis A, Cassibba V, Mattei D, Gianfaldoni G, Scattolin AM, Di Bona E, Oldani E, Parolini M, Gokbuget N, Bassan R (2013) High cure rates in Burkitt lymphoma and leukemia: a Northern Italy Leukemia Group study of the German short intensive rituximab-chemotherapy program. Haematologica 98 (11):1718-1725. doi:10.3324/haematol.2013.086827

6. Hoelzer D, Walewski J, Dohner H, Viardot A, Hiddemann W, Spiekermann K, Serve H, Duhrsen U, Huttmann A, Thiel E, Dengler J, Kneba M, Schaich M, Schmidt-Wolf IG, Beck J, Hertenstein B, Reichle A, Domanska-Czyz K, Fietkau R, Horst HA, Rieder H, Schwartz S, Burmeister T, Gokbuget N, German Multicenter Study Group for Adult Acute Lymphoblastic L (2014) Improved outcome of adult Burkitt lymphoma/leukemia with rituximab and chemotherapy: report of a large prospective multicenter trial. Blood 124 (26):3870-3879. doi:10.1182/blood-2014-03-563627

7. Wildes TM, Farrington L, Yeung C, Harrington AM, Foyil KV, Liu J, Kreisel F, Bartlett NL, Fenske TS (2014) Rituximab is associated with improved survival in Burkitt lymphoma: a retrospective analysis from two US academic medical centers. Therapeutic advances in hematology 5 (1):3-12. doi:10.1177/2040620713514682

8. Mead GM, Barrans SL, Qian WD, Walewski J, Radford JA, Wolf M, Clawson SM, Stenning SP, Yule CL, Jack AS, Cli UNCRIL, Lymphoma AL (2008) A prospective clinicopathologic study of dose-modified CODOX-M/IVAC in patients with sporadic Burkitt lymphoma defined using cytogenetic and immunophenotypic criteria (MRC/NCRI LY10 trial). Blood 112 (6):2248-2260. doi:DOI 10.1182/blood-2008-03-145128

9. Wasterlid T, Brown PN, Hagberg O, Hagberg H, Pedersen LM, D'Amore F, Jerkeman M (2013) Impact of chemotherapy regimen and rituximab in adult Burkitt lymphoma: a retrospective population-based study from the Nordic Lymphoma Group. Ann Oncol 24 (7):1879-1886. doi:10.1093/annonc/mdt058

10. A predictive model for aggressive non-Hodgkin's lymphoma. The International Non-Hodgkin's Lymphoma Prognostic Factors Project (1993). The New England journal of medicine 329 (14):987-994. doi:10.1056/NEJM199309303291402

11. Mead GM, Sydes MR, Walewski J, Grigg A, Hatton CS, Norbert P, Guarnaccia C, Lewis MS, McKendrick J, Stenning SP, Wright D, Collaborators UL (2002) An international evaluation of CODOX-M and CODOX-M alternating with IVAC in adult Burkitt's lymphoma: results of United Kingdom Lymphoma Group LY06 study. Ann Oncol 13 (8):1264-1274. doi:DOI 10.1093/annonc/mdf253

12. Jacobson C, LaCasce A (2014) How I treat Burkitt lymphoma in adults. Blood 124 (19):2913-2920. doi:10.1182/blood-2014-06-538504

**Supplemental table**

| **Supplemental Table 1 Prognostic factors** | | | | | |
| --- | --- | --- | --- | --- | --- |
| Variables |  | 5-year OS | HR | 95% CI | P-value |
| Age  continuous  < 40 years // ≥ 40 years |  | -  79% / 60% | 1.06  2.3 | 1.03-1.09  1.11-4.72 | <0.001  0.025 |
| Gender M // F |  | 71% / 69% | 1.037 | 0.488-2.203 | 0.925 |
| Ann Arbor stage  I-II // III-IV |  | 81% / 67% | 1.83 | 0.70-4.77 | 0.216 |
| Extranodal involvement  bone marrow |  | 75% / 63% | 1.58 | 0.77-3.26 | 0.212 |
| Extranodal involvement  central nervous system |  | 74% / 60% | 1.63 | 0.77-3.46 | 0.205 |
| Extranodal involvement  gastrointestinal tract |  | 76% / 63% | 1.83 | 0.90-3.71 | 0.093 |
| Extranodal involvement  ≥ 2 sites |  | 78% / 61% | 2.05 | 0.97-4.30 | 0.059 |
| Bulky disease ≥ 10 cm  no / yes |  | 73% / 58% | 1.83 | 0.86-3.89 | 0.116 |
| LDH  normal / elevated |  | 81% / 65% | 1.90 | 0.73-4.95 | 0.188 |
| Peripheral blood blasts ≥ 30%  no / yes |  | 69% / 100% | ^a^ | ^a^ | ^a^ |
| HIV positivity  no / yes |  | 71% / 69% | 1.15 | 0.40-3.33 | 0.791 |
| WHO performance score  0-1 / 2-4 |  | 69% / 68% | 0.98 | 0.43-2.21 | 0.963 |
| Year of diagnosis, continuous |  | - | 0.95 | 0.87-1.04 | 0.243 |
| IPI-score  low (0-2) / high (3-5) |  | 81% / 65% | 2.46 | 1.13-5.35 | 0.023 |
| BL score  Low (0) / high (1-4) |  | 81% / 67% | 1.77 | 0.42-7.43 | 0.434 |
| Rituximab in regimen  yes / no | all  < 40 years  ≥ 40 years | 74% / 55%  80% / 73%  67% / 33% | 1.91  1.50  2.33 | 0.88-4.14  0.41-5.53  0.88-6.14 | 0.103  0.545  0.088 |
| Treatment group  LMB  BFM  HOVON  CODOX-M/IVAC | all | 67%  74%  72%  71% | -  0.80  0.95  0.91 | -  0.28-2.28  0.39-2.31  0.34-2.41 | 0.981  0.680  0.903  0.843 |
| Treatment group  LMB  BFM  HOVON  CODOX-M/IVAC | < 40 years | 77%  78%  80%  82% | -  1.01  1.01  0.78 | -  0.20-5.19  0.24-4.22  0.15-4.02 | 0.991  0.994  0.991  0.767 |
| Treatment group  LMB  BFM  HOVON  CODOX-M/IVAC | ≥ 40 years | 50%  70%  64%  60% | -  0.55  0.76  0.86 | -  0.14-2.12  0.24-2.40  0.25-2.93 | 0.849  0.383  0.640  0.805 |
| ^a^ could not be calculated due to lack of events | | | | | |

**Supplemental Fig. 2:** (a) Overall A survival of BL patients < 40 years of age treated with the LMB, BFM, HOVON or CODOX-M/IVAC regimens. (b) Overall survival of BL patients ≥ 40 years of age treated with the LMB, BFM, HOVON or CODOX-M/IVAC regimens. (c) Overall survival of BL patients with an IPI-score of 0-2 treated with the LMB, BFM, HOVON or CODOX-M/IVAC regimens. (d) Overall survival of BL patients with an IPI-score of 3-5 treated with LMB, BFM, HOVON or CODOX-M/IVAC regimens. L MB: black line, BFM: red line, HOVON: green line, CODOX-M/IVAC: blue line


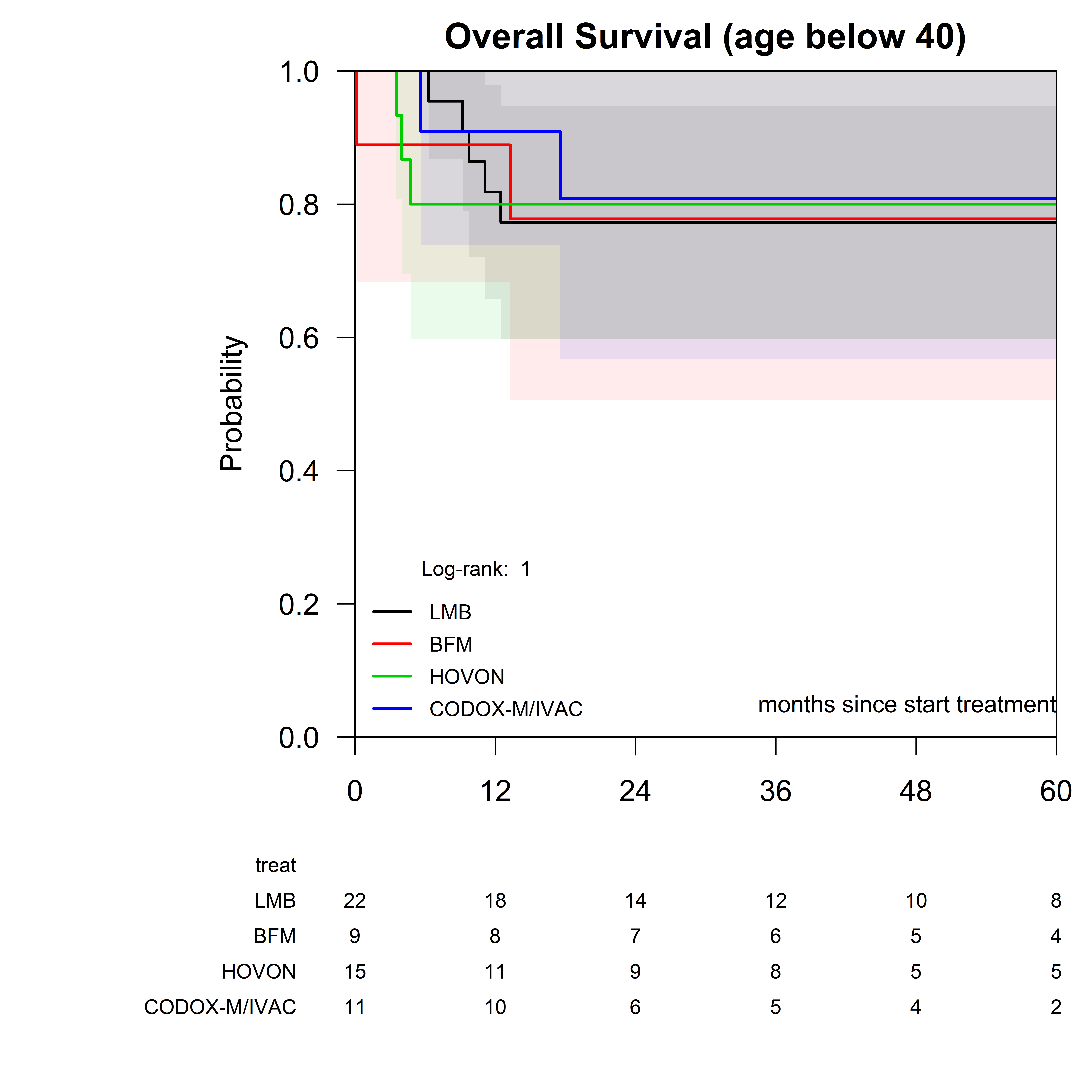

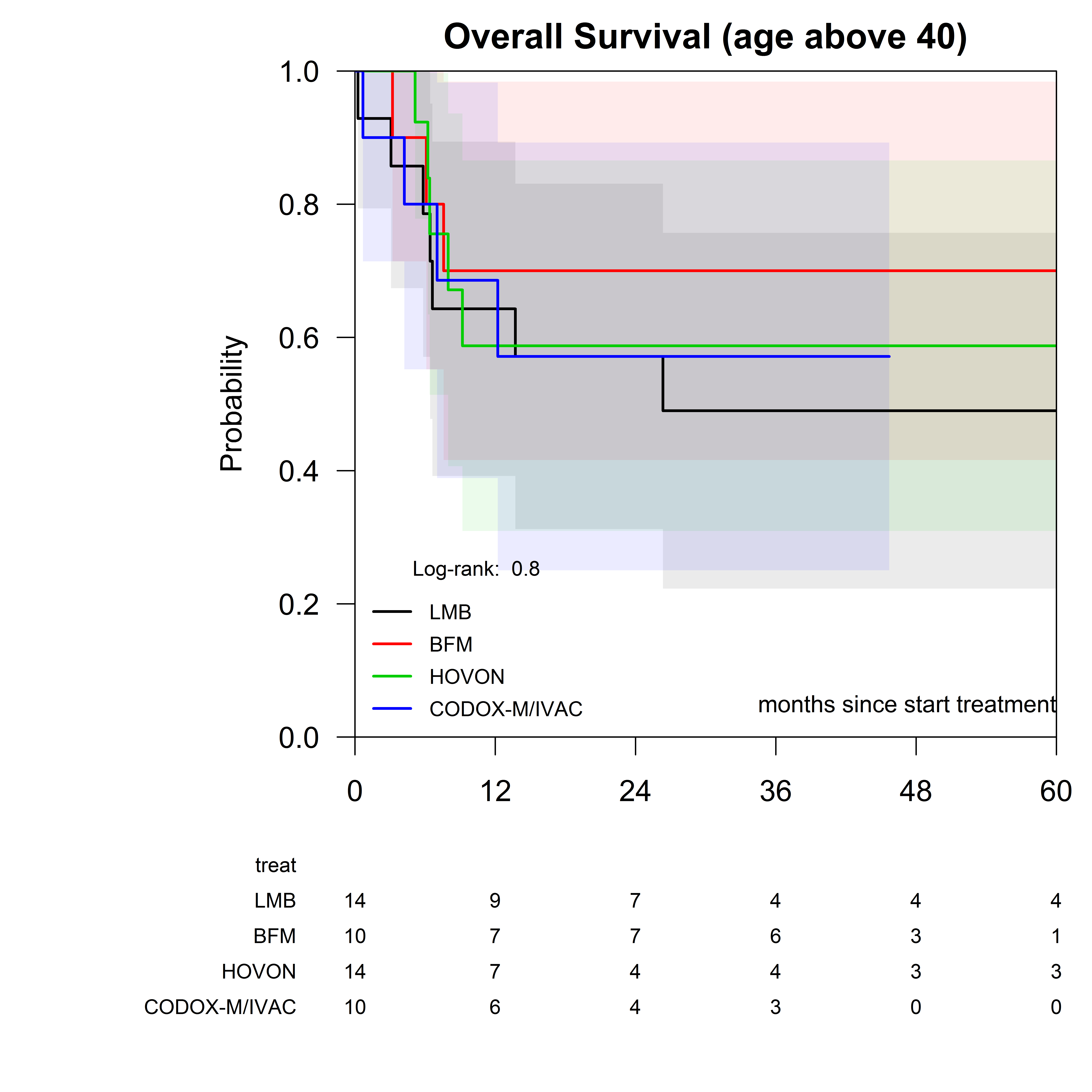

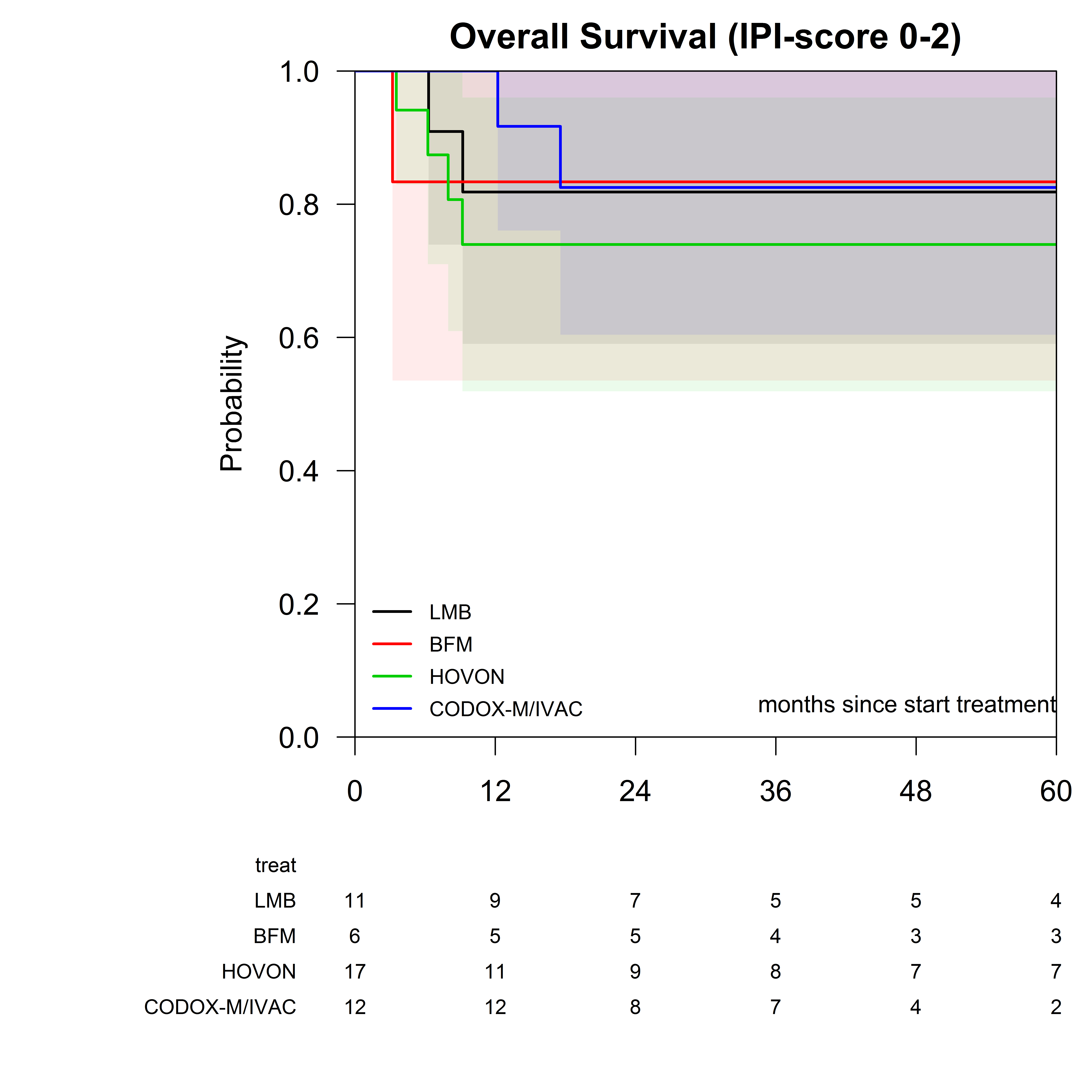

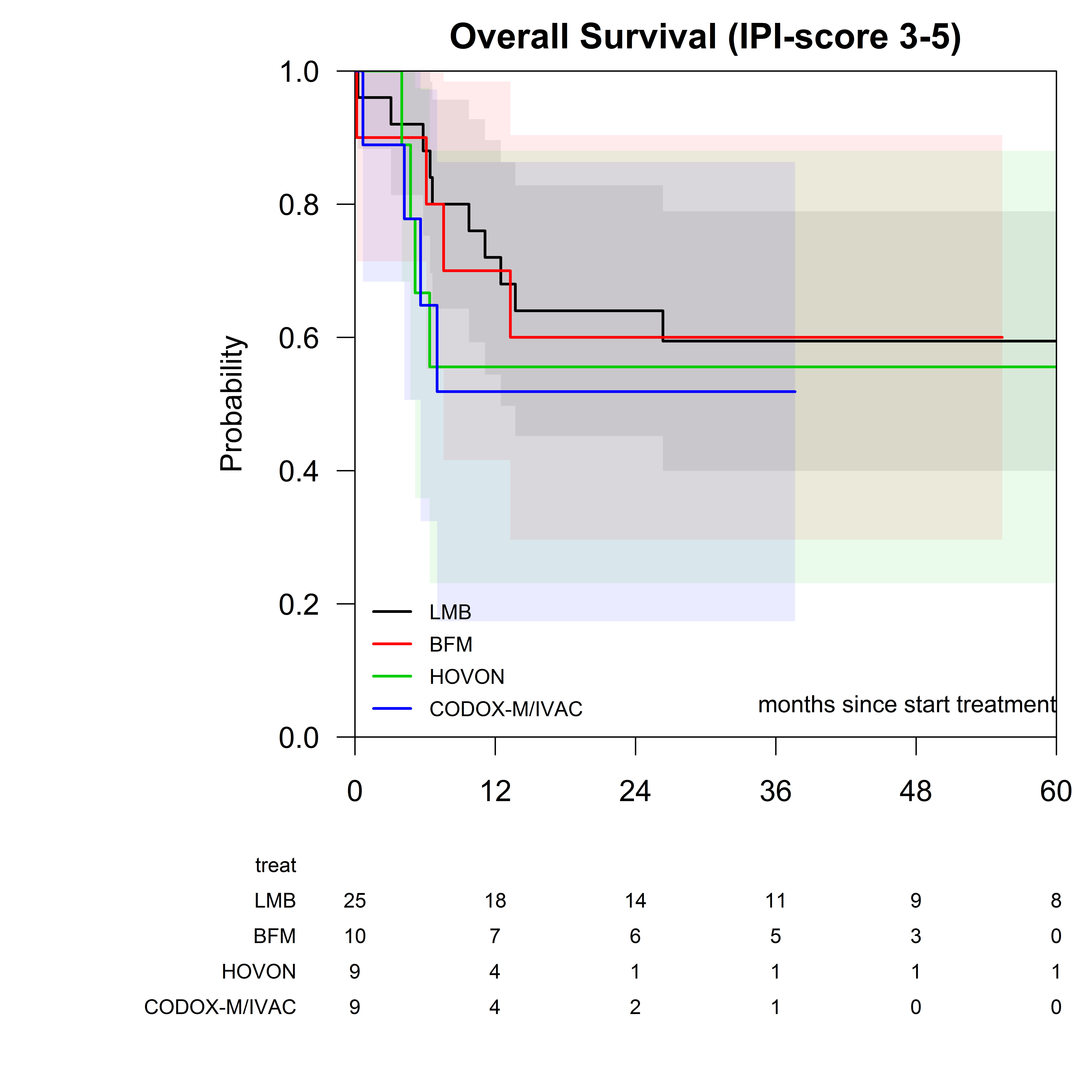


a

b

c

d
